# Multimeric Stability of Human C-reactive Protein in Archived Specimens

**DOI:** 10.1371/journal.pone.0058094

**Published:** 2013-03-14

**Authors:** Qiling Li, Ting Kang, Xiaohua Tian, Yamin Ma, Min Li, Jendai Richards, Tameka Bythwood, Yueling Wang, Xu Li, Dong Liu, Li Ma, Qing Song

**Affiliations:** 1 Department of Obstetrics and Gynecology, First Affiliated Hospital, Xi'an Jiaotong University, Xi'an, Shaanxi, China; 2 Cardiovascular Research Institute, Morehouse School of Medicine, Atlanta, Georgia, United States of America; 3 School of Information Science and Engineering, Central South University, Changsha, China; The University of Texas MD Anderson Cancer Center, United States of America

## Abstract

**Background:**

C-reactive protein (CRP) is a marker of inflammation and a risk predictor of cardiovascular disease. Current CRP assays are focused on the quantification of the CRP levels as pentamers. However, CRP can be present as other multimeric forms. There will be a market need to measure the CRP multimeric structure in addition to the levels in human populations. To meet this need, we investigated whether the long-term archived samples could be used instead of freshly collected samples.

**Methodology/Principal Findings:**

The specimens of serum, plasma and tissues were collected from transgenic rats expressing the human CRP. These samples were stored at 4°C, −20°C and −80°C for different periods. Non-denaturing Western blot analysis was used to observe the influence of storage conditions to multimeric structures of human CRP. Our results showed that there was no difference on multimeric structures of human CRP between samples stored at 4°C, −20°C and −80°C, between samples stored at −80°C for twenty-four hours and three months, and between plasma and serum.

**Conclusions/Significance:**

This study implicated that archived samples stored at these conditions in those large longitudinal studies could be used for investigating the multimeric structures of CRP. Our report may speed up these researches and save labors and budget by enabling them to use currently available archived samples rather than freshly collected samples.

## Introduction

C-reactive protein (CRP) is a member of the pentraxin protein family. CRP is an ancient and ubiquitous protein in vertebrates and invertebrates. CRP was originally found in the plasma of patients with acute infections, as a substance that reacted with the C polysaccharide of the pneumococcus [1], [2]. It is a classical marker for systemic inflammation and tissue injury as an acute phase reactant; the level of CRP in the plasma may rise by 1000-fold or more during inflammation.

Beside its role as a sensitive inflammation marker, CRP has shown values on prediction of disease risk and decision making on treatment for a series of diseases. It has been well known that baseline CRP concentration has strong predictive and prognostic values for future cardiovascular events [3], [4], [5]. The Centers for Disease Control and Prevention and the American Heart Association reported that it is reasonable to measure CRP as an adjunct to the measurement of established risk factors in order to assess the risk of coronary heart disease [6]. High CRP levels might indicate the benefit from statin therapy [7], [8]. In addition to cardiovascular disease and stroke, elevated CRP concentration was found to be associated with other diseases, such as diabetes [9], [10], colon cancer [11]. For example, patients with colon cancer have significantly higher CRP concentrations in their blood than the individuals without colon cancer [11]. Baseline CRP levels increase with the stage of β-cell dysfunction and insulin resistance [12]. A very high value of CRP is a strong parameter for the diagnosis and the treatment of several diseases, such as cell giant arteritis (also called temporal arteritis or Horton's disease) [13], [14], vasculitis Takayasu disease [15], [16] and polymyalgia rheumatica [17], [18]. Serum CRP levels are helpful in the prediction of prognosis and coronary involvement of the patients with Kawasaki disease, when considering age [19], [20]. A large population study revealed an age-dependent relationships among serum CRP levels, diagnostic sub-categories and prognosis in the Kawasaki patients [20]. The role of CRP has been implicated in the pathogenesis of age-related macular degeneration (AMD) by immunohistochemical evidence that showed clear changes in distribution and relative levels of CRP and complement factor H (CFH) in early and late AMD eyes [21]. Synergistic influence of CRP levels and the at risk genotype of the CFH gene resulted in a super-additive risk for prevalent late AMD and AMD progression [22]. These studies have demonstrated the value of CRP in the pre-symptom risk predictions of human diseases.

**Figure 1:**
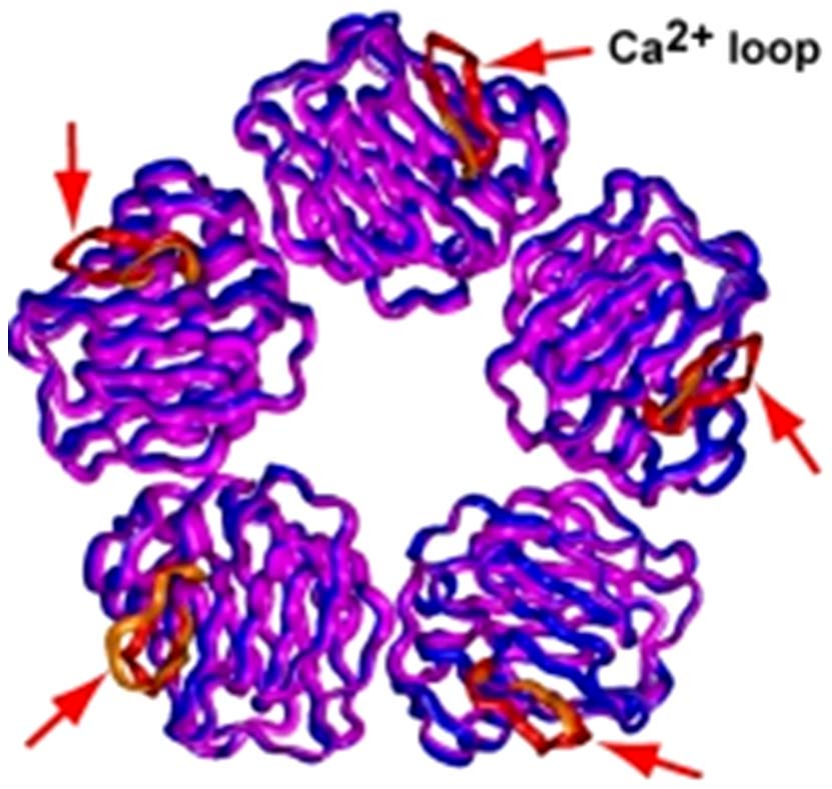
Pentameric structure of human C-reactive protein, cited from [**46**].

CRP is synthesized by the liver and then secreted into the blood [7]. The CRP protein contains 206 amino acids including an 18-amino acid signal sequence [23], [24]. CRP can be present as a pentamer in a cyclic structure with 5 identical 23-kD subunits in the blood (Fig. 1) [7], or as a monomer in the intimal region of blood vessels in normal human tissues and the atherosclerotic plaque [25], [26], [27]. The elevation of CRP concentration may be caused by a rise of interleukin-6 (IL-6) and tumor necrosis factor alpha (TNF-alpha) produced predominantly by macrophages [7] as well as adipocytes [28]. It has been shown that diet may play a role in the CRP concentration, individuals in the highest quartile of trans-fat consumption had blood levels of CRP that were 73% higher than those in the lowest quartile [29]. The CRP molecule has both a recognition and an effector function [30]. CRP has an ability to recognize specifically foreign pathogens and damaged cells of the host. It has been proposed that one of its major physiologic functions is to act as a scavenger for chromatin released by dead cells during the acute inflammatory process [31]. Pentameric CRP may bind to large ligands containing phosphocholine via a phosphate oxygen that projects away from the surface of the protein. In this process, one planar face of the CRP pentameric molecule binds to a phosphocholine-bearing surface, the opposite exposed face may serve as the recognition site for C1q for complement activation [32]. It has been reported that pentameric CRP (pCRP) and monomeric CRP (mCRP) showed some opposite functions [26], [33], [34], [35], [36]. In addition, we recently found that CRP may be present in other isoforms in other tissues, such as trimers and tetramers. There may be a need to measure the CRP multimeric structure in addition to the high-sensitivity baseline CRP levels.

Because freshly collected samples are not always available in many large and longitudinal epidemiological studies, it is important to know if these valuable archived specimens could be used to study the CRP multimeric isoforms. For example, the Framingham Heart Study is a long-term and ongoing cardiovascular study on residents of Framingham, Massachusetts [37], the Jackson Heart Study is a large study to investigate the genetic factors of cardiovascular disease in African-Americans [38]. In the Framingham Heart Study, the serum samples were collected as early as 1940s from individuals of different generations and frozen specimens have been archived since then. The serum or plasma samples have been used to measure the CRP concentration in clinical and epidemiological studies. However, it is unknown if these valuable specimens could be used to study the polymorphism of CRP multimeric isoform and its association with human diseases. In this study, we examined whether multimeric structure of human CRP were well preserved after storage.

## Materials and Methods

### Samples

The serum, plasma, and tissues containing human CRP were obtained from transgenic rats (7–52 weeks old) expressing human CRP. Rats were genotyped with 50 ng of genomic DNA extracted from the tail biopsies by PCR using the primers (5′-ACA TAC GCA AGG GAT TTA GTC-3′, 5′-AAC AGC TTC TCC ATG GTC AC-3′). All transgenic rats were anesthetized with 3% isoflurane by standard surgical procedures before collection of blood. From each rat, we collected about 2 ml of whole blood in 10-ml glass tubes (BD vacutainer tubes 367874) with or without sodium heparin. From this 2-ml blood from each rat, 1-ml was used for obtaining serum, the other 1-ml was used for obtaining plasma. Then serum and plasma were both split into three aliquots. For evaluating assay performance across sample types, matched sets of specimens of two types (serum and plasma) were collected simultaneously. The contents of collection tubes without anticoagulant were allowed to clot at room temperature for 1 hour. Tubes were centrifuged at 3,000 g for 15 minutes at room temperature. Totally, 14 rats were used in this study, four rats at 9-weeks old, one rat at 7-weeks old, 2 rats at 14-weeks old, one rats at 18-weeks old, one rat at 30-weeks old, 4 rats at 37-weeks old, one rat at 52-weeks old.

After the transgenic rats (Rat-A, 9-week old; Rat-B, 37-week old) were anesthetized with 70% CO_2_, liver, pancreas, fat, kidney, aorta, heart and muscle were collected. Harvested tissues were snap frozen in liquid nitrogen. Protein was extracted with T-PER Tissue Protein Extraction Reagent (Thermo Scientific, Prod# 78510), with the Halt protease inhibitor cocktail, EDTA-free (100×) (Thermo Scientific, Cat. No. 87785). About 30 mg of tissues was homogenized with a Teflon glass homogenizer in an extraction medium (1 volume protease inhibitor cocktail in 29 volume tissue protein extraction reagent) of a 10-fold volume of the pellet. After centrifugation at 4°C for 10 min, an aliquot of the supernatant was used to determine the protein concentrations with BCA assay in Biophotometer (Eppendorf).

All animal experiments were performed by the approval of the Institutional Animal Care and Use Committee of Morehouse School of Medicine, and conformed to the Guide for the Care and Use of Laboratory Animals published by the National Institutes of Health.

### Sample storage

To determine the influence of storage temperature on CRP stability, serum and plasma samples were split into three aliquots (each aliquot for each storage temperature) and stored at 4°C, −20°C, and −80°C, respectively, for twenty-four hours and three months. All tissues including liver, pancreas, adipose, kidney, aorta, heart and muscle were removed at termination, split into two aliquots and stored at −80°C for twenty-four hours and three months. The samples were then brought to room temperature and analyzed for CRP.

### Western Blot for CRP

Protein concentrations were determined using the Biophotometer (Eppendorf). Totally 20 ug of total proteins were heated at 70°C for 10 min to prevent protein aggregation, electrophoresized on a 4–12% non-denaturing polycrylamide gels and transferred to PVDF membranes. Membranes were blocked with 5% milk for 1 hour, and then incubated with anti-human CRP antibody overnight. The membranes were washed 3 times with 1×TBST (1% serum albumin in 50 mM Tris-HCl pH 7.4, containing 0.05% Tween-20) at room temperature, and then incubated with goat anti-mouse IgG-HRP antibody (Santa Cruz, Sc-2005, 1∶2,000 dilution) at room temperature for 1 hour. Membranes were washed 3 times and then incubated with substrate solution (SuperSignal West Pico Chemilluminescent Kit) for 5 minutes and exposed to X-ray film. Three independent anti-human CRP antibodies were used in these Western blot analysis, M86284M (Meridian), M86005M (Meridian), and ab52687 (abcam).

## Results

### Influence of storage period on the CRP multimeric structure

**Figure 2:**
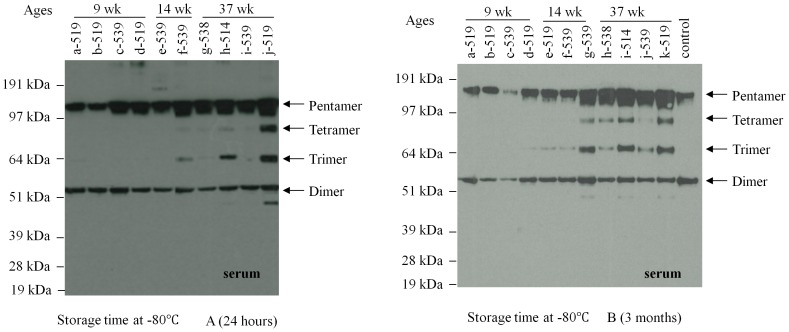
The influence of serum storage time on the multimeric structure of CRP. The serum samples were stored at −80°C for twenty-four hours or three months. Western blot showed that the serums from the same rat displayed the similar multimeric structure of CRP after they were stored at −80°C for a different period. A) Serum stored for twenty-four hours at −80°C. B) Serum stored for three months at −80°C. The specificity of the antibody is shown by a serum sample from a non-transgenic rat.

The serum samples that were stored at −80°C for either twenty-four hours or three months were analyzed by Western Blot. These samples were obtained from rats at different ages (4 rats of 9 weeks old, 2 rats of 14 weeks old, and 4 rats of 37 weeks old), so they appeared to have multiple isoforms in their serum. As shown in Figure 2, the results from samples stored for twenty-four hours and stored for three months displayed no significant difference, indicating that the time length of the storage at −80°C did not affect significantly the preservation of CRP multimeric structure in the specimens. No monomer has been detectable in the non-denaturing Western blot analysis.

Although this paper presents the data from 14 rats, they are representatives from totally 76 young rats and 43 senior rats. We have scanned the band intensities of the trimers and tetramers with the Image J software and calculated the statistical significance between junior and senior rats on the appearance of the trimers and tetramers. The mean intensity of junior rats is 0.93±1.03, the mean intensity of senior rats is 148.69±48.76, the P value is 0.0127.

**Figure 3:**
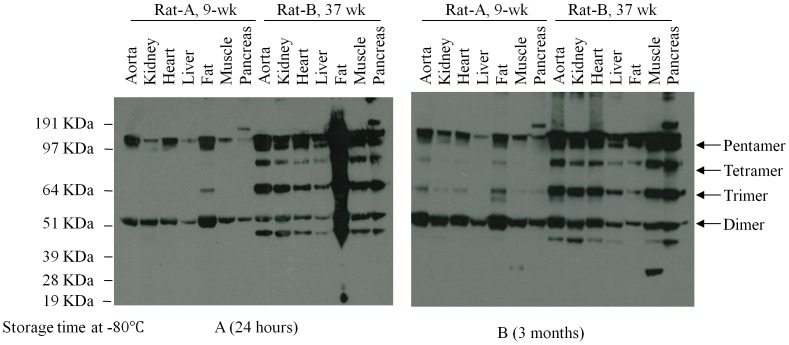
The influence of time length of storage on the multimeric structure of CRP. The samples were stored at −80°C for twenty-four hours or three months. Western blot showed that the samples from the same tissue of the same rat displayed the similar multimeric structure of CRP after they were stored at −80°C for a different period. A) Samples stored for twenty-four hours at −80°C. B) Samples stored for three months at −80°C.

The tissue specimens of human transgenic CRP rats (9-weeks old and 37 weeks old) were analyzed by Western Blot (Fig. 3). Tissue samples were taken from the aorta, kidney, heart, liver, fat, muscle and pancreas. All samples were stored at −80°C for twenty-four hours and three months. The ages of rats were 9 weeks and 37 weeks for rat A and rat B respectively. There was no significant difference in the multimerization of human CRP at twenty-four hours and three months.

### Influence of storage temperature on the CRP multimeric structure

**Figure 4:**
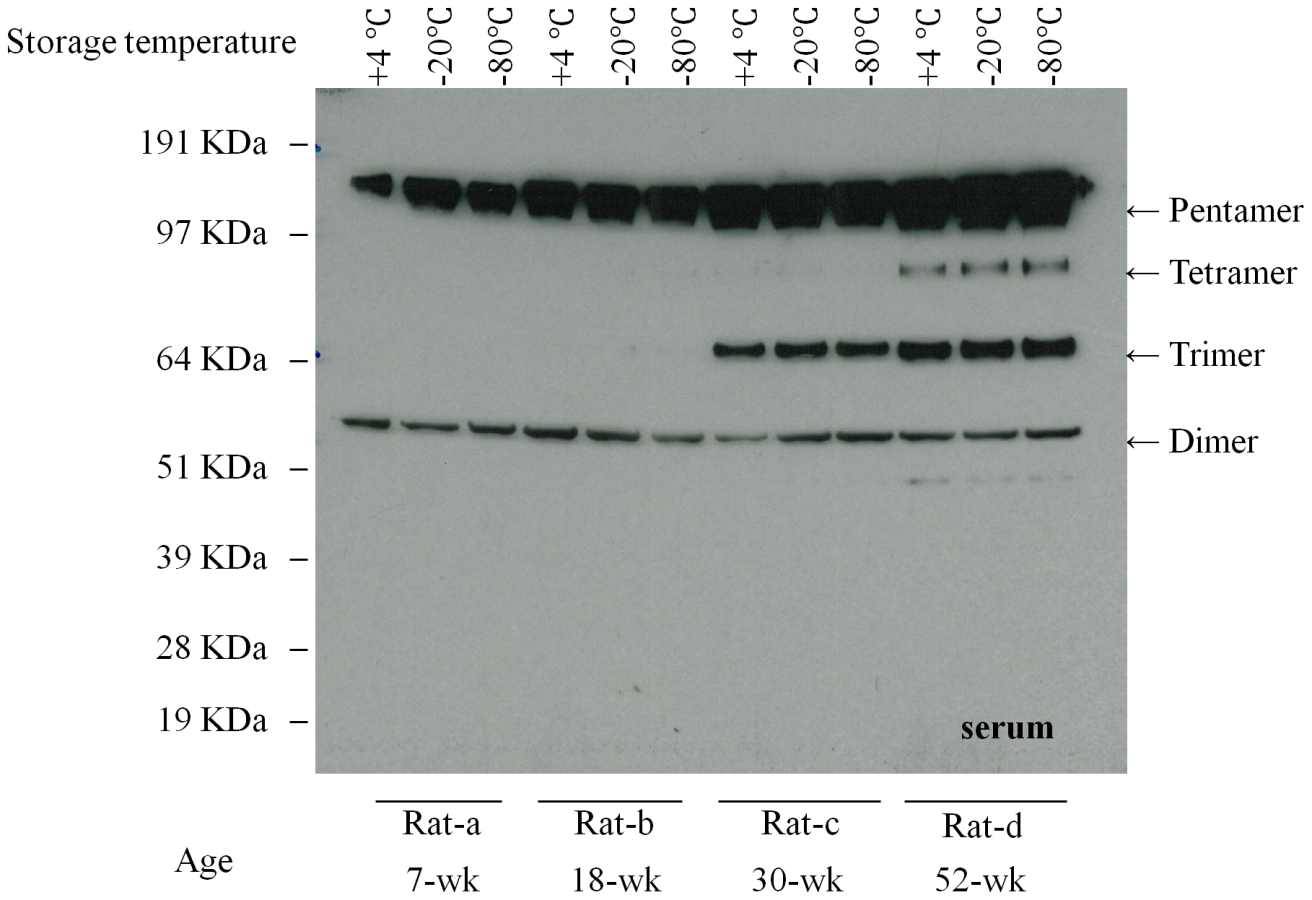
The influence of storage temperature on the multimeric structure of CRP. The serum samples were stored at different temperature for three months. Western blot was performed. There is no significant difference on the multimeric isoforms between the samples from the same rats stored at different temperature.

The serum samples that were stored at different temperature for three months were analyzed by Western Blot. In young rats (7 weeks old and 18 weeks old), human CRP was present in serum as both pentamers and dimers. Trimers and tetramers were well preserved in the old rats (30 weeks old and 52 weeks old). There was no significant difference in CRP concentrations between samples stored at 4°C, −20°C and −80°C for three months (Fig. 4). No other isoforms were detected by non-denaturing Western blot gels and there was no aggregation or degradation of dimers or pentamers in the serum.

### Influence of sample type on the CRP multimeric structure

**Figure 5:**
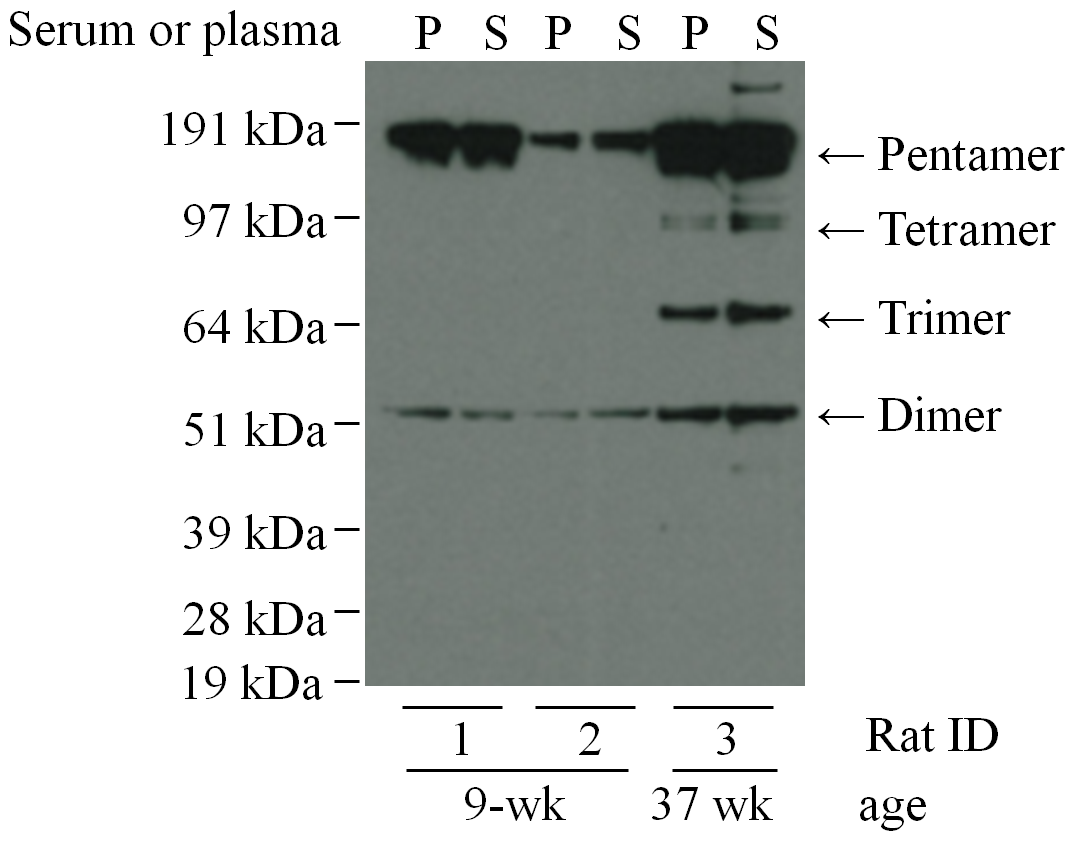
Multimeric structure of human CRP in plasma and serum. After being stored at −80°C for three months, plasma and serum (diluted as 1∶1000) of same transgenic human CRP rats were examined for expression of human CRP by non-denaturing Western blot analysis. Rat No.1 and No. 2 were 9-week old, rat No. 3 was 37-week ages. P: plasma, S: serum.

Plasma and serum of the same transgenic human CRP rats were examined by Western Blot after storage at −80°C for three months (Fig. 5). In the young Rat1 and Rat2 (9 weeks old), both serum and plasma showed only pentamer and dimer isoforms. In the senior Rat-3 (37 weeks old), both the pentamers, tetramers, trimers, and dimers were well preserved in the serum and plasma specimens. There was no significant difference on the observed CRP structure between plasma and serum in the same rat.

## Discussion

This study assessed the stability of multimeric structure of human C-reactive protein in archived specimens of blood and tissues. Our results showed that the multimeric structure of human CRP were well preserved after storage at 4°C, −20°C, and −80°C for a period of three months. These data indicated that the archived human samples in human cohorts could be used to examine the CRP structure polymorphisms in addition to simply measuring the concentrations among human populations.

The conformation of CRP may be critically related to the functions of CRP. The physiological role of CRP is to bind to phosphocholine expressed on the surface of dead or dying cells (and some types of bacteria) in order to activate the complement system via the C1q complex [32]. It has been reported that mCRP showed opposite biological functions compared with pCRP. For example, mCRP induced interleukin-8 secretion in neutrophils [39] and human coronary artery endothelial cells [34], promoted neutrophil-endothelial cell adhesion [40], and delayed apoptosis of human neutrophils [41]. Monomeric CRP, but not pentameric CRP, accumulated in human atherosclerotic lesions [27]. It was suggested that mCRP might be the active isoform that plays a direct role in atherogenesis via modulating the behavior of the monocytes [42]. Protein aggregation is a common feature of many neurodegenerative diseases, including Alzheimer's, Parkinson's, Huntington's diseases, amyotrophic lateral sclerosis, frontal temporal dementia, and the human prion diseases [43]. It is assumed that the aggregation process plays a central role in pathogenesis. In these diseases, misfolding of a particular protein can lead to its aggregation, involving a process in which monomers interact to form dimers, oligomers, and eventually insoluble fibrillar deposits [44], [45]. Further studies will be needed to clarify whether other CRP isoforms have distinct physiological functions compared with pentameric CRP.

In summary, current assays on CRP measurement are focused on highly sensitive quantification of the CRP levels. However, recent evidence suggested that CRP multimeric structure should be considered in addition to the CRP levels when investigating its pathological role and predictor value in cardiovascular disease and other diseases [26], [27], [33], [34], [35], [36], [42]. This study assessed the stability of multimeric structure of human C-reactive protein in archived specimens of serum and tissues. Our results showed that the multimeric structure of human CRP were well preserved after storage at 4°C, −20°C, and −80°C for a period of three months.
